# Effects of an Alternative Sports Program Using Kin-Ball in Individuals with Intellectual Disabilities

**DOI:** 10.3390/ijerph17155296

**Published:** 2020-07-23

**Authors:** Félix Zurita-Ortega, José Luis Ubago-Jiménez, Pilar Puertas-Molero, Irwin Andrés Ramírez-Granizo, José Joaquín Muros, Gabriel González-Valero

**Affiliations:** Department of Didactics of Musical, Plastic and Corporal Expression, University of Granada, 18071 Granada, Spain; felixzo@ugr.es (F.Z.-O.); jlubago@ugr.es (J.L.U.-J.); pilarpuertas@correo.ugr.es (P.P.-M.); irwinrg@ugr.es (I.A.R.-G.); jjmuros@ugr.es (J.J.M.)

**Keywords:** physical activity, intellectual disability, Kin-Ball, motor skills, intervention program

## Abstract

The first aim of the present work was to examine the effects of a physical activity sports program, specifically Kin-Ball, within a group of individuals with intellectual disabilities, on decreasing sedentary behavior and improving basic physical skills. The second aim was to evaluate social validity and acceptability of the intervention. In this pre-experimental study, 47 individuals participated (46.8% male and 53.2% female) with an average age of 29.85 (SD = 10.41). All participants were administered an intervention program based on the alternative sport of Kin-Ball. BMI was calculated for body composition and age-related Z-scores were interpreted with the tables provided by the WHO. Endurance was measured through a modified six-minute test, speed was analyzed using a 50 m test, and strength was estimated according to a hand-grip dynamometer. Likewise, balance and coordination were examined in line with adaptations proposed by the scientific literature. Results indicated that all cases experienced statistically significant differences following the intervention program (*p* = 0.000). Improvement effects were detected in all post-intervention tests (endurance, strength, speed, balance, and coordination). As a main conclusion, it is indicated that an alternative sports-based program improves physical ability and motor skills in individuals with intellectual disabilities.

## 1. Introduction

Intellectual disabilities (IDs) are understood as those conditions characterized by significant limitations in both intellectual functioning and adaptive behaviors. Depending on the level of intellectual disability, there is a consensus that describes delays in motor functioning [1]. For a disorder to be considered as an ID, it must present itself before an individual reaches 18 years of age [2,3]. Such disabilities are reported by 3% of the world’s population according to data provided by the World Health Organization (WHO) [4]. In Spain, this figure is estimated to incorporate more than 400,000 people [5], which is highly significant considering that this type of population experiences related effects on various ambits of life development (health, education, and leisure).

As is logical, the characteristics presented by populations with IDs are accentuated with increasing age and add to those that are derived from aging [6,7]. Thus, a series of characteristics can be highlighted which pertain to these disabilities and include: poor balance (which can lead to falls in advanced age), poor motor skills and coordination problems, high obesity levels (such individuals are four times more likely to be overweight than populations without disabilities), and depleted attention and concentration capacities [7,8,9].

Regular engagement in physical activity (PA) is one of the factors which has been seen to demonstrate quality of life improvements within the general population. Such practice is importance for the maintenance of health in all of its dimensions [10,11,12]. Nevertheless, as has been stated by Einarsson et al. [13], disparities exist between the PA levels of individuals with and without disabilities. Other authors [14,15] have indicated that individuals with these conditions are less likely to engage in sufficient levels of PA, with this leading to high levels of sedentary behavior and potentially provoking health problems. Likewise, with increasing age and the transition to adolescence, the quantity and intensity of physical activity engaged in by individuals with IDs reduces further still [16,17]. To this end, participation in sport or physical activity can provide important health benefits at a physical, psychological, and social level, alongside equivalent improvements in quality of life, as has been stated by Luiselli [18,19]. Furthermore, scientific evidence exists that indicates that engagement in PA can improve levels of motivation, empathy, and social skills. Increasing levels of sport practice and reducing sedentary behavior, amongst individuals with disability, could have a favorable impact on their health given the numerous benefits highlighted which include: increased circulation, decreased dependence, reduced risk of suffering cardiovascular diseases, type 2 diabetes, arterial hypertension, obesity, and osteoporosis [9]. Likewise, Ogg-Groennedaal et al. [20] and Johnson [21] stated that individuals with intellectual disabilities who are more active are better equipped to combat osteoporosis, osteoarthritis, thus, provoking improvements in balance, strength, endurance, and flexibility, as well as reductions in overweight indices. In addition, PA can also improve health indirectly. For example, sport practice improves the physical cardiorespiratory state, which, at the same time, decreases mortality risk, even in the presence of overweight or obesity [22,23].

These studies are corroborated by authors such as Boddy et al. [24] or Pérez-Cruzado and Cuesta-Vargas [25], who showed significant improvements in the quality of life of individuals who were administered an intervention which incorporated recreational physical activities. Other intervention programs that use similar methodologies also showed benefits of PA engagement at a physical, psychological, and social level in children with disabilities [26,27,28,29]. A study conducted by Ulrich et al. [30] stands out as they obtained positive results through a program which taught participants to ride a bicycle. Following their intervention, improvements were seen in the moderate-vigorous physical activity level of recipients. Likewise, studies conducted with adults or older adults, such as that carried out by Shields [31], Melville et al. [28], and Van Schinjdel-Speet et al. [32], have had success. They administered a step program and produced improvements at a physical level in terms of increased cardiovascular endurance, coordination, and balance.

Van Schijndel-Speet et al. [32], administered an intervention based on karate and showed improved motor and psychosocial development within individuals with IDs. Studies conducted with accelerometers and young people with IDs have also been highlighted. These studies have objectively quantified habitual PA through sporting practice (playing petanque) and the time spent engaged in sedentary pursuits. Such methods are reliable and practical as they do not pose any demands on the cognitive capacity of the individuals being monitored, making them especially suitable for evaluating sporting practice [33,34].

A particularly useful sport in this regard is Kin-Ball (alternative sport). This sport stands out because of its dynamic characteristics and emphasis on teamwork, cooperation, and the sporting spirit of those involved. Studies such as those performed by Hall et al. [35], demonstrated, from a coeducational standpoint, that practice of these types of sports increased motivation amongst the least active. As shown by Hastie et al. [36], Kin-Ball kept participants of different activity categories more active as compared with other sporting disciplines. According to the International Kin-Ball Sport Federation [37], three teams are formed with four players on each team. The main objective is to control and defend the ball before it touches the ground, as points are scored each time the opposing team commits a foul. The team that maintains possession of the ball is in charge of hitting the ball, with all team members being in contact with the ball. Specifically, if the defending team does not control the ball before it hits the ground, each of the other two teams will receive a point.

Physical education teachers of students with special educational needs need to know about the active strategies that they can use with the class as a whole. This is important as the majority of implementation programs are undertaken as case studies or with individual students. Although research on this topic is highly limited, some authors [38,39] have suggested that physical activity and behavioral interventions with complete groups could be useful for preventing disassociation among individuals with intellectual disabilities and promoting positive behaviors amongst the whole group. Others such as Thomson et al. [40] provided, through their work, a collection of studies relating to intellectual disabilities and intervention through physical activity. Thus, the purpose of the present study was to examine the effects of a physical activity sports program using Kin-Ball within a group of individuals with intellectual disabilities, on decreasing sedentary behavior and increasing basic physical capacity (endurance, speed and strength, balance, and coordination).

## 2. Materials and Methods

### 2.1. Participants and Procedures

**Figure 1:**
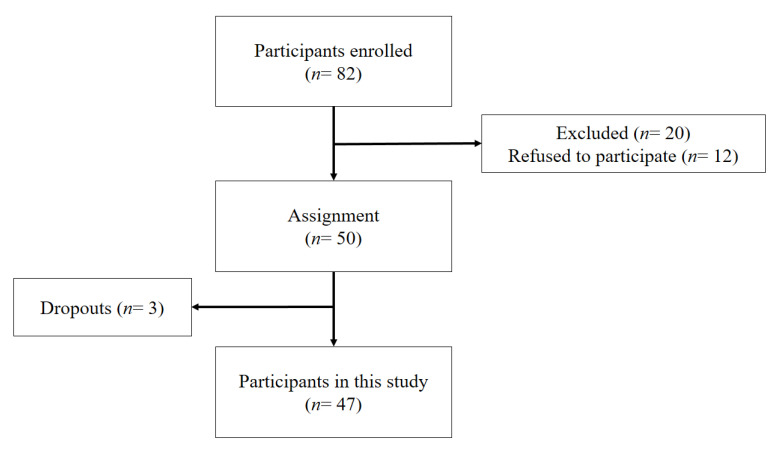
Flowchart of participants.

The legal guardians of participants gave informed consent for participation and the procedure applied was approved by the Committee of Research Ethics at the 1230/CEIH/2020. A total of 47 individuals (46.8% male and 53.2% female) participated in this pre-experimental study. Participants were attending a Special Education Centre and had an average age of 29.85 years (SD = 10.41), with age ranging from 12 to 55 years. The Special Education Center has 13 units of Basic/Primary Education and four relating to the Training Program for Transition to Adult Life and Work. All participants attended 12 sessions, with data being collected at the beginning and end of the program. Participants were referred to the program by supervisors and workers at the aforementioned center as part of the physical activity plan they were receiving. Inclusion criteria for the present study stipulated that participants must have been diagnosed with an intellectual disability; however, all other center users were also invited to participate in a voluntary way. Participant flow through the phases of the intervention program is shown in Figure 1.

### 2.2. The Kin-Ball Program

Table 1 shows activities of the Kin-ball intervention program. The Kin-Ball program was adapted in order to maintain the essential objectives of teaching and learning inherent in this alternative sport [36,41]. In order to maintain didactic commitment, researchers undertook the relevant training needed to enable them to comply with the standards necessary to impart this new sport with individuals with intellectual disabilities.

The first adaptation involved two sessions which sought to increase knowledge and initiate contact with students, those responsible for their physical education and support staff. This ensured that these individuals had initial contact with this sporting modality. Likewise, informal meetings were held at the end of each session with the main working and auxiliary staff. Here, queries were raised around those questions that could be improved or changed. This helped to clarify ideas and establish new tasks and additional activities which would lend greater adaptability to the sport.

A second adaptation was made in which the traditional 8 weeks were extended to 12 weeks in order to enable greater familiarity with the activities and contents of this modality. Each session lasted 1 h in real time, with participants arriving 20 min prior to session initiation and leaving after 1 h of the activity had passed.

Program exercises were adapted for the first few sessions but later, and then throughout the program, a sequencing of this sport modality was developed [42]. The medical reports of the participants were reviewed to consider possible musculoskeletal and physical sports problems during the intervention. Adapted exercises were introduced throughout in response to individual needs (different learning and execution rates). Some of the innovations in additional practice which arose were to help participants generalize their practice. Another modification included the use of pictograms or cartoons to increase participants’ capacity to internalize practice. The adaptations and intervention processes were supervised by center staff and the authors of this research.

### 2.3. Variables and Instruments

The study was centered on health-related components of physical aptitude and followed the categories proposed by the American College of Sports Medicine (ACSM), i.e., body composition, muscular strength, and endurance [43]. Flexibility was not included in accordance with the recommendations proposed by Ganley et al. [44]. Firstly, relevant literature was reviewed to determine the most appropriate tests. Secondly, the tests to be used were agreed upon with implicated physical education and physiotherapy teachers. To this end, these aforementioned individuals discussed the potential problems inherent with each test (functionality, and cognitive and motor demands). Finally, a pilot study was developed which was conducted with a sample of 14 users and reached maximum reliability in the fourth week (intraclass correlation >0.7). From these, a number of tests were selected for the present study, namely, body mass index (BMI), modified 6 min walking test to determine endurance, dynamometer test to evaluate strength, 50 m test to determine speed, and static balance and the tap test to determine coordination. An open question was also included, asking participants to report their perceptions of the activity. This question was written by the caregiver or staff member in charge of the activity and was responded to individually.

#### 2.3.1. Body Composition

BMI was calculated as body weight measured in kg divided by height in meters squared. Age-related Z-scores were calculated for BMI according to growth charts stipulated by the WHO. Then, these Z-scores were classified as underweight when zBMI was <2 SD. For children younger than 6 years, overweight was classified when zBMI was >2 SD and obesity was determined in cases in which zBMI was >3. For those aged between 6 and 18 years, zBMI scores >1 were classified as overweight and >2 as obese [45]. The WHO standards for BMI in older adults were applied and categorized according to the following: underweight (below 18.5), healthy weight (18.5–24.9), overweight (25.0–29.9), and obese (30.0 and above). High test-retest reliability was found, with significant results related to BMI for children with a moderate or severe ID (Interclass Correlation Coefficients (ICC) > 0.99).

#### 2.3.2. Physical Qualities

Endurance was measured through a modified 6 min test which has been validated within populations with intellectual disabilities [46]. Speed was analyzed using a 50 m test, and strength was estimated according to a hand-grip dynamometer, following the adaptations proposed by Boer and Moss [47]. Likewise, balance and coordination were examined in line with adaptations proposed by Skowroński, Horvat et al. [1].

### 2.4. Procedures

Both pre- and post-intervention tests were conducted individually in a sports hall, with each session following a fixed order, i.e., weight, height, manual dynamometry, coordination, balance, speed, and endurance. Participants were evaluated by the same professionals for the pre- and post-intervention tests. Specifically, the supervision and evaluation team included occupational therapists, physical education graduates, and physical therapists. This sequence meant that the easiest tests (body composition and dynamometry) were completed at the start, progressing towards the most difficult test which was resistance. A rest was allowed between performance of the various tests. A 12-week intervention program (one session per week) was developed. All individuals participated on the same day, although they were divided into three groups according to age groups. Two of the groups had a total of 16 participants, while the other group had 15 participants.

Evaluators explained activities to each participant individually and performed demonstrations. Participants were encouraged to do their best at all times and to complete tasks in the correct fashion. No standardized instructions were given, because each student had their own personal preferences and communication styles. Instead, instructors were permitted to use their judgement with regard to if the participant had understood the way in which they were expected to perform the tests, with maximum effort and in accordance with the protocol. All instructors had prior experience with the testing procedures and were experts working with children with intellectual disabilities.

### 2.5. Data Analysis

A descriptive analysis was carried out in order to describe participants’ characteristics. Pre- and post-intervention test differences among groups were determined through Chi-square and Pearson analysis, whereas independent t-tests were used for interval data. Results for a number of the tests were shown through descriptive statistics. The magnitude of differences (effect sizes) was obtained while using the standardized measure Cohen’s d (*d*) interpreted as follows: null (0–0.19), low (0.20–0.49), moderate (0.50–0.79), or high (≥0.80) [48]. A confidence interval to 95% for each effect sizes was calculated. Data were analyzed using SPSS version 24.0, with alpha being set at 5%. Normality and homogeneity of the sample were examined through the Kolmogorov–Smirnov test, all variables were normally distributed (*p* ≥ 0.05).

## 3. Results

Table 2 shows descriptive analysis. Data, from the present study, show that there was a total of 47 participants (53.2% females (*n* = 25) and 46.8% males (*n* = 22)) with an average age of 29.85 years (SD = 10.411). Average fat mass was 21.51 kg (SD = 9.97), fat-free mass was 47.25 kg (SD = 8.45), and total water mass was 33.14% (SD = 6.12). With regards to the initial data obtained (pre-intervention stage), the following results were obtained in the various conducted tests: BMI (M = 25.22 and SD = 4.46), strength (M = 19.38 and SD = 6.10), speed (M = 11.25 and SD = 2.09), endurance (M = 478.73 and SD = 76.81), coordination (M = 70.57 and SD = 19.14), and balance (M = 24.31 and SD = 19.74).

Following this, correlations among variables collected in the pre-intervention stage were examined (see Table 3). It was seen that no associations were established among any of the variables, apart from between balance and speed (*r* = −355).

Table 2 shows the results recorded after implementation of the intervention program (post-intervention stage) and indicates the following data: BMI (M = 25.07 and SD = 4.38), strength (Str) (M = 21.06 and SD = 5.95), speed (Spe) (M = 10.66 and SD = 2.11), endurance (End) (M = 500.70 and SD = 72.24), coordination (Coo) (M = 85.68 and SD = 19.67), and balance (Bal) (M = 32.53 and SD = 20.36). With regards to correlations among variables collected during the post-intervention stage, data showed that no association was uncovered among the variables (see Table 4).

Finally, analysis of data designed to uncover the effects of the intervention program based on Kin-Ball indicated that statistically significant differences were found in all cases (*p* = 0.000), with improvements being detected in all of the post-intervention stage tests (Table 2). Cohen’s d effect size showed a null effect for BMI (d = 0.034, and CI −0.538, 0.606); low effect for strength (d = 0.279 and CI −0.296, 0.853); speed (d = 0.281 and CI −0.294, 0.856); endurance (d = 0.295 and CI −0.280, 0.870); balance (d = 0.410 and CI −0.168, 0.988); and moderate effect for coordination (d = 0.779 and CI 0.186, 1.372).

## 4. Discussion

The present study indicates that individuals with intellectual disabilities have low levels of health-related physical aptitude. However, the 47 participants who participated in a physical activity intervention program through Kin-Ball experienced improvements in their physical abilities.

Other studies delivered with similar samples around the world have obtained positive values similar to those reported by the present study and have had positive effects on motor development [49,50], although the fact that such effects decrease with age [51], must be considered. The sample receiving the present intervention had similar characteristics to those reported in other studies with regards to sex, fat mass, fat free mass, and total water mass. The other studies involved participants with the same characteristics as stated in their reviews [52].

Initial baseline data, collected in the first stage prior to any intervention, showed similar values to those found in studies such as those conducted by Bazzano et al. [53], Harris et al. [54], Jeng et al. [55], and Kong et al. [56]. These authors carried out intervention studies with similar populations and also found inferior values to those measured for their peers without any disability. Nonetheless, following development of respective interventions, it can be confirmed that physical exercise is a useful strategy for maintaining and improving physical condition.

A moderate correlation was found between speed and balance, i.e., those with better balance employed less time to complete the speed test. This agrees with the fact that an individual with an intellectual disability occasionally suffers problems in relation to balance, musculoskeletal issues, and decreased muscle tone [57]. This impacts upon their physical capacity to carry out tasks, their potential for being physically active, and, more specifically, results in impaired stability leading to a reduced capacity when they participant in running races.

Physical activity programs for individuals with intellectual disabilities are fairly abundant and consider certain aspects to be highly positive. Over recent years, aerobic [50,58], mindfulness [59], swimming [60], and Tai-Chi [55] programs have stood out; however, no program has incorporated an alternative sport such as Kin-Ball. Thus, the particular characteristics of this modality (physical, cognitive, and inclusive) had remained unexplored.

Implementation of the program revealed improvements in all post-intervention stage tests. This establishes that, when performed in a continuous way, Kin-Ball is beneficial for the physical and social development of those with intellectual disabilities [41,61]. Indeed, its practice is associated with a lower risk of overweight and obesity, and a reduced likelihood of suffering from cardiovascular diseases, hypertension, or other metabolic pathologies. These are some of the main aims to be considered within this population group [62,63].

In relation to intervention, effects based on this sporting modality have demonstrated improvements in intention, motivation, and attitude, with these all being important psychological factors considered by the model proposed by Van der Ploeg et al. [64].

Despite the fact that the individuals with intellectual disabilities who participated in the present study had some type of musculoskeletal and physical-sporting problem [57], it was possible to achieve improvements in general physical state, physical activity, as well as skills and motor skills [65]. Sessions lasting between 45 and 60 min produced improvements in the motor functioning of individuals with severe and profound intellectual disabilities [43]. Daytime specialist centers in Spain are not required to organize physical education sessions, in contrast to what occurs in the case of regular education, which is of course mandatory. Given the poor state of physical aptitude seen within this vulnerable population, the government and relevant institutions must conduct regular physical examinations in order to ensure that developed programs do, in fact, mitigate for low physical activity levels.

## 5. Conclusions

Limitations of the present research study exist, by the fact that it was developed from within a group of participants formed by 82 children and adolescents who were identified has having moderate to severe disabilities. Nonetheless, the sample size included only 47 participants as participants abandoned some tests, in some cases as a result of a lack of adaptive behavior or poor motor development. Future studies should include larger samples and consider the socioeconomic status and background of individuals. This is important given that previously conducted studies have demonstrated that these variables are also associated with a good physical state [66,67]. The reference values used also have limitations given that standardized values were used for age. Another important element to consider is that gender differences appear during puberty [68].

The main conclusions obtained through evaluation of the adequacy of a physical activity program based on Kin-Ball are that all participants increased their speed, endurance, strength, coordination, and balance. Participants also reported high levels of satisfaction and motivation towards this alternative sport.

## Figures and Tables

**Table 1:** List of activities of the Kin-ball intervention program.

Characteristics	Session					
	1–2	3–4	5–6	7–8	9–10	11–12
Activity	Tag	Tag	Wall	Tag	Wall	Wall
	Relay run	Omnikin	Indiana Jones	Relay run	Together	Tag
	Centipede	Together	Centipede	Indiana Jones	Tripod	Indiana Jones
	Together	Relay run	Tripod	Omnikin	Omnikin	Omnikin
	Tripod	Match	Match	Match	Match	Match
Development	Centipede: Lying on the floor they pass the ball					
	Indiana Jones: A person within the circle of players avoiding being touched by the ball					
	Match: Kinball match simulations					
	Omnikin: Ball hits with hands					
	Relay run: Ball handling in couples and trios					
	Tag: Touching a partner with the Omnikin ball					
	Together: At the signal “together” they have to touch the ball					
	Tripod: Holding the ball among three people					
	Wall: One player with the ball blocks others from passing from one zone to another					

**Table 2:** Results for pre- and post-intervention tests.

Variables	M	SD	Standard Error Average	Sig	ES (d)	95% CI
BMIpre	25.22	4.468	0.651	0.000	0.034	(−0.538, 0.606)
BMIpost	25.07	4.385	0.639			
Strepre	19.38	6.101	0.890	0.000	0.279	(−0.296, 0.853)
Strepost	21.06	5.958	0.869			
Balpre	24.31	19.748	2.880	0.000	0.410	(−0.168, 0.988)
Balpost	32.53	20.363	2.970			
Spepre	11.25	2.099	0.306	0.000	0.281	(−0.294, 0.856)
Spepost	10.66	2.118	0.308			
Endpre	478.73	76.816	11.204	0.000	0.295	(−0.280, 0.870)
Endpost	500.70	72.244	10.537			
Coopre	70.57	19.142	2.792	0.000	0.779	(0.186, 1.372)
Coopost	85.68	19.670	2.869			

Note 1: Body mass index pre (BMIpre); strength pre (Strpre); speed pre (Spepre); endurance pre (Endpre); balance pre (Balpre); coordination pre (Coopre); body mass index post (BMIpost); strength post (Strpost); speed post (Spepost); endurance post (Endpost); balance post (Balpost); coordination post (Coopost). Effect sizes (ES); confidence interval (CI).

**Table 3:** Correlations pre-intervention tests.

Variables	IMCpre	Strpre	Balpre	Spepre	Endpre	Coopre
BMIpre	1.000					
Strpre	−0.136	1.000				
Balpre	−0.066	0.187	1.000			
Spepre	0.075	−0.160	−0.355 *	1.000		
Endpre	−0.242	0.046	−0.064	−0.009	1.000	
Coopre	0.197	0.111	0.011	−0.050	0.106	1.000

Note 1: Body mass index Pre (BMIpre); strength pre (Strpre); speed ore (Spepre); endurance pre (Endpre); balance pre (Balpre); coordination pre (Coopre). The bilateral correlation is significant at the 0.05 level (*).

**Table 4:** Correlations post-intervention tests.

Variables	BMIpost	Strpost	Balpost	Spepost	Endpost	Coopost
BMIpost	1.000					
Strpost	−0.079	1.000				
Balpost	−0.147	−0.003	1.000			
Spepost	0.149	−0.109	−0.279	1.000		
Endpost	−0.205	0.133	−0.142	0.076	1.000	
Coopost	0.073	0.105	0.073	−0.157	0.196	1.000

Note 1: Body mass index post (BMIpost); strength post (Strpost); speed post (Spepost); endurance post (Endpost); balance post (Balpost); coordination post (Coopost).
